# Tracking Adaptation and Fidelity When Embedding COPE, Evidence-Based Dementia Care, in PACE Sites

**DOI:** 10.1093/geroni/igab046.664

**Published:** 2021-12-17

**Authors:** Nancy Hodgson, Laura Gitlin

**Affiliations:** 1 University of Pennsylvania, School of Nursing, Philadelphia, Pennsylvania, United States; 2 Drexel University, College of Nursing and Health Professions, Drexel University, Pennsylvania, United States

## Abstract

One essential question in moving dementia care interventions to practice is, “What is the optimal balance between fidelity to, and adaptation of, a proven program in “real world” settings?" We present a protocol for measuring the adaptation/fidelity and implementation of an evidence-based dementia care program (Care of Persons in their Environment, COPE) in PACE settings. During pre-implementation, science-based elements of COPE were documented including the theory of change, logic model and core components. Possible adaptations to COPE in its delivery were identified and included program structure (sequence of sessions), content (assessments), and delivery methods (online). During implementation, documentation of implementation strategies is captured using an evidence-informed checklist derived from the Expert Recommendations for Implementing Change (ERIC) workgroup. Ongoing documentation of fidelity/adaptation aspects of program implementation is conducted using the FRAME framework. Understanding methods and measures deployed in adaptation and implementation of evidence-based dementia programs can help guide future translation efforts.

